# Unveiling barriers and prioritizing strategies for empowerment of Oraon tribal women through multi-criteria decision analysis

**DOI:** 10.3389/fsoc.2026.1707192

**Published:** 2026-05-19

**Authors:** Seema Kujur, Sukanya Barua, Manjeet Singh Nain, Subhashree Sahu, V. Sangeetha, Anirban Mukherjee, K. V. Praveen, Nuthaki Venkata Leela Krishna Chaithanya, Girish Kumar Jha

**Affiliations:** 1 Division of Agricultural Extension, ICAR-Indian Agricultural Research Institute, New Delhi, India; 2 Division of Agricultural Extension, ICAR-Research Complex for Eastern Region, Patna, India; 3 Division of Agricultural Economics, ICAR-Indian Agricultural Research Institute, New Delhi, India; 4 Division of Bioinformatics, ICAR-Indian Agricultural Statistics Research Institute, New Delhi, India

**Keywords:** AHP, economic inclusion, empowerment, gender equality, India, Oraon tribal women

## Abstract

**Introduction:**

Tribal women play a vital role in their communities, often managing both economic and domestic responsibilities. Despite the growing feminization of agriculture in India, women continue to face multifaceted challenges in accessing and controlling resources, exercising agency, and technological advancements, which collectively impede their empowerment and the achievement of SDG 5. These constraints are more pronounced among Oraon tribal women, who experience compounded disadvantages due to historical marginalization.

**Objective:**

This study identifies key challenges and formulates strategic interventions for empowering Oraon tribal women in Jharkhand and Chhattisgarh, India.

**Materials and methods:**

The cross-sectional study was carried out to assess challenges to women's empowerment. A total of 400 women of reproductive age were selected to prioritize challenges, and 40 officials were randomly selected to formulate the strategies using the Analytic Hierarchy Process for empowerment.

**Results:**

The Oraon tribal women revealed five critical barriers: socio-cultural (domestic violence, unequal household burdens), economic (wage disparities, limited employment), educational (poverty-driven dropout rates), technical (lack of agricultural knowledge), and psychological (low self-efficacy). Economic and educational challenges emerged as the most significant, with 72.89% and 63.12% severity scores, respectively. AHP-based prioritization highlighted top strategies including economic inclusion through financial services (weight: 0.326) and land access (weight: 0.249), educational investments in infrastructure (weight: 0.332) and quality schooling (weight: 0.227) mass awareness campaigns against domestic violence (weight: 0.298); residential agricultural training (weight: 0.336) and gender sensitization programs (weight: 0.272).

**Conclusion:**

The study underscores the need for integrated policies addressing economic participation, education access, and community engagement to dismantle systemic barriers. Findings align with global SDG5 targets, advocating for women-centered extensions and government initiatives to enhance decision-making autonomy. This research provides a scalable framework for tribal women's empowerment, emphasizing multi-criteria decision-making in development planning.

## Introduction

Empowerment has become a central focus within the global development agenda, particularly under the framework of the Sustainable Development Goals (SDGs). At the heart of this is SDG 5, which explicitly aims to “achieve gender equality and empower all women and girls.” This goal underscores the transformative potential of empowerment through its nine specific targets and 14 measurable indicators (Elias et al., 2021). Women are a vital part of the economy, and they constitute 40% of the total labor force globally. Over the years, there has been a gradual realization of the key role of women in agricultural development and their vital contribution in the fields of agriculture, food security, horticulture, processing, nutrition, sericulture, fisheries, and other allied sectors. Women contribute significantly to the world's food supply and remain integral to farming communities across the globe. Recognizing and strengthening their participation is therefore essential for the effective design and implementation of programs aimed at enhancing agricultural productivity and sustainability (Ghosh et al., 2021; Gopal and Vijayabhinandana, 2012; Sharma, 2022). In India and many other developing countries, rural women constitute a substantial proportion of the workforce. Women are the backbone of almost every element of agriculture. Women have made major contributions to agriculture, from spreading seeds to selling agricultural products. In rural India, women constitute 84% of the workforce, with 73% of them employed in agriculture (Sharma, 2022). Given women's substantial contributions to agriculture and allied sectors, women's empowerment is a precondition for the nation's development and women are empowered when they become more self-aware, autonomous, economically successful, politically engaged, and capable of making well-informed judgments on issues that impact them (Mokta, 2022). Although it is essential, the path to women's empowerment is frequently hampered by several issues, including cultural attitudes, financial limitations, and violence. Cultural ideas might often cause women to inadvertently obstruct other women's advancement. Inadequate educational attainment often sustains superstitious beliefs, which may manifest in unethical social behavior, and despite making up 43% of the agricultural workforce, women frequently lack access to and control over resources and autonomy (Divya et al., 2025; Sharma, 2022).

Compared to other countries , India has a larger percentage of tribal people (Manohar et al., 2025). The tribal population, which makes up only 8.6% of India's total population, is primarily distributed in seven states (Odisha, Jharkhand, Madhya Pradesh, Maharashtra, Chhattisgarh, Gujarat, and West Bengal) and the northeastern region (NER, which includes Arunachal Pradesh, Assam, Manipur, Meghalaya, Mizoram, Nagaland, Tripura, and Sikkim). The majority of these states are dominated by indigenous (tribal) populations (Verma et al., 2025). The Oraon is an agricultural tribal community and one of the largest tribal groups in India; it constitutes about 3.6 million people in the country's total population. Oraon tribe is a Dravidian-speaking ethnolinguistic group inhabiting to Chhota Nagpur Plateau and adjoining areas, mainly the Indian states of Jharkhand, Odisha, Chhattisgarh, and West Bengal. They are also inhabiting other states, viz. Madhya Pradesh, Maharashtra, and Bihar (Tirkey and Jain, 2006; Chandra and Paswan, 2020). Their tribal language is known as Kurukh (Dravidian), but they are also fluent in Sadri, Odia, Hindi, Bengali, and Marathi (Chandra and Paswan, 2020). Despite being considered the original inhabitants of the land, tribes are not allowed access to basic requirements. Along with their deficiencies in education, society, and the economy, they also face various types of discrimination, especially among women (Manohar et al., 2025). The challenges, including poverty, exploitation, displacement, land alienation, illiteracy, and a lack of health-care services, faced by adivasis affect both men and women. The government provides people with monetary and social structural benefits, but the monetary benefits do not ensure their equality in society, and such grants are not part of social integration (Maitra, 2016). The Oraon tribe, *one* of India”s largest Scheduled Tribes, exemplifies this struggle: despite constituting 3.6 million people, Oraon women endure compounded marginalization due to socio-cultural norms, economic deprivation, and limited institutional support (Chandra and Paswan, 2020; Government of India, 2011).

Women's empowerment and gender discrimination exhibit significant disparities across cultural, regional, and national contexts (Elias et al., 2021). In India, although the Constitution and government legislation guarantee women equal rights with men, tribal women are still striving for full equality and continue to experience significant disparities in social and economic freedoms compared to their male counterparts and to women in urban areas (Thakur and Kumar, 2024; Naveen et al., 2023). In patriarchal societies, such as India's tribal communities, women often face systemic barriers rooted in traditional gender roles, which restrict their autonomy, economic participation, and access to education (Mokta, 2022; Mal and Saikia, 2024). Globally, 43% of the agricultural workforce in developing nations comprises women, yet they rarely enjoy equitable access to resources or decision-making power (Quisumbing et al., 2023). In India, tribal women face a literacy rate of just 41% (compared to 59% for tribal men), while 73% engage in agricultural labor without land ownership or fair wages (Sharma, 2022; Manohar et al., 2025). For Oraon women, these challenges are exacerbated by early marriages, domestic violence, and limited access to technology (Divya et al., 2025). Despite their pivotal role in sustaining agrarian economies, their contributions remain undervalued, perpetuating cycles of disempowerment (Ghosh et al., 2021). Therefore, there was a critical need for systematic empirical evidence to understand the extent and dimensions of these challenges and their implications for women's empowerment.

This study addresses these gaps by identifying the multidimensional challenges (socio-cultural, economic, educational, technical, and psychological) hindering Oraon women's empowerment, and prioritizing evidence-based strategies using the Analytic Hierarchy Process (AHP) to inform policy interventions. By centering on tribal women's lived experiences, this research aligns with SDG5's mandate to “achieve gender equality and empower all women and girls” (United Nations, 2015).

## Materials and methods

### Analytical framework

**Figure 1:**
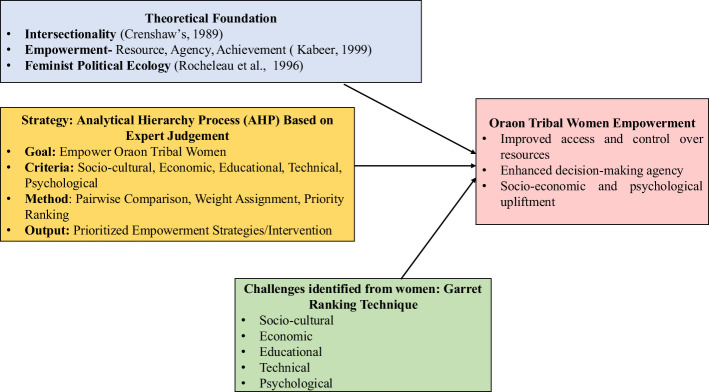
Analytical framework.

This study adopts an intersectional lens to explore how gender, tribal identity, and socio-economic status collectively intensify the challenges faced by Oraon women, drawing on foundational work on intersectionality (Crenshaw, 1989). The analytical framework is rooted in the theory of empowerment (Kabeer, 1999), which conceptualizes empowerment through three interrelated dimensions: resources, agency, and achievements. Within this framework, structural barriers such as patriarchal norms (Mokta, 2022) and systemic economic exclusion (Sen, 1999) are identified as key factors limiting women's access to education, land ownership, and participation in decision-making processes. To prioritize strategic interventions, the study employs the Analytic Hierarchy Process (AHP) developed by Saaty (1980), a multi-criteria decision-making tool that ranks factors across socio-cultural, economic, educational, technical, and psychological dimensions. This aligns with the principles of Feminist Political Ecology, which emphasizes gendered access to and control over resources (Rocheleau et al., 1996). The framework is operationalized as follows: resources are represented by land ownership and educational access (Kabeer, 1999); agency includes participation in self-help groups and financial autonomy (Malhotra et al., 2002); and achievements are measured by indicators such as reduced domestic violence and increased literacy, in line with Sustainable Development Goal 5. By integrating theoretical and methodological approaches, this model provides a robust, replicable structure for analyzing empowerment in tribal settings (Figure 1).

### Sampling plan

**Figure 2:**
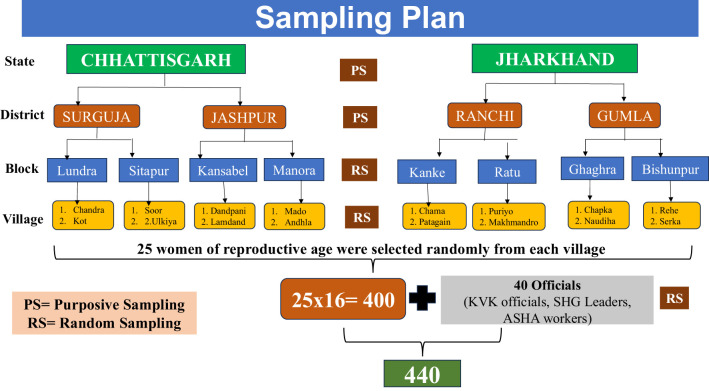
Sampling plan of the study.

The cross-sectional study was conducted in the eastern and central regions of India, with Chhattisgarh and Jharkhand purposively selected based on their significant tribal populations, particularly of the Oraon community. Chhattisgarh and Jharkhand have a high proportion of tribal population, accounting for 30.6 and 26.2% of their total populations, respectively. Moreover, these states have a high concentration of the Oraon tribal population, with 7,48,789 in Chhattisgarh and 17,16,618 in Jharkhand (Census, 2011). Within each state, two districts with high Oraon concentration were purposively selected namely Jashpur (2,74,224), and Surguja (2,85,885) in Chhattisgarh, and Ranchi (5,30,287) and Gumla (4,42,659) in Jharkhand, totalling four districts. From each district, two blocks were randomly selected, namely Lundra & Sitapur in Surguja, and Kansabel & Manora in Jashpur. Similarly, Kanke & Ratu in Ranchi and Ghaghra & Bishunpur in Gumla, totalling eight blocks. From each block, two villages were randomly selected, followed by a total of 25 Oraon women of reproductive age group (15-49 years) were randomly selected from each village, resulting in a sample of 400 respondents for prioritizing the severity of challenges. Additionally, to inform the development of strategic interventions, at the district level, two KVK personnel from each KVK were randomly selected, comprising eight KVK personnel, and at the village level, from each village, one SHG leader and one ASHA worker were randomly selected, which constitutes 32 officials at the village level. So, totalling it with KVK personnel makes it 40 officials who are engaged in field-based work with tribal communities to enhance the representativeness of the sample of the stakeholders and to elicit the holistic strategic intervention. In this way, the total sample size to 440 respondents for the study as mentioned in Figure 2.

## Data and statistical methods

### Prioritization of challenges by Oraon tribal women

To systematically identify and prioritize the most frequently encountered challenges faced by Oraon tribal women, the Garrett Ranking Technique (Garett and Woodworth, 1969) was employed. This method was chosen for its robustness in converting qualitative assessments into quantitative scores, thereby enabling the ranking of challenges based on their perceived severity. The technique involves calculating mean scores for each identified challenges and assigning ranks accordingly, offering a clear understanding of the relative importance of each issue from the respondents' perspective. It is extensively used in social science research to systematically transform qualitative data into quantitative measures, thereby facilitating rigorous comparison and analysis. Additionally, this technique organizes the factors according to respondents' preferences (Ranjan et al., 2025; Radhika et al., 2023).

### Steps of Garrett ranking

**Step 1: Identification of factors/dimensions:** Through an extensive review of literature, expert consultation, and field-level interactions during pretesting, a set of key challenges was identified. These were classified into five dimensions: socio-cultural, economic, educational, technical, and psychological.

**Step 2: Collection of ranks from respondents:** Respondents were asked to rank the challenges based on their perceived severity, ranging from most severe to least severe.

**Step 3: Conversion of ranks into percent positions:** The ranks given by respondents are converted into percent positions using the following formula:


$$\begin{array}{l} \text{Percent position}=\frac{100\;\left(Rij-0.05\right)}{Nj} \end{array}$$

Where,

R_ij_ = Rank given for the i^th^ challenge by the j^th^ respondent.

N_j_ = Number of challenges ranked by the j^th^ respondent

**Step 4: Conversion of percent positions into Garrett scores:** The calculated percent positions were converted into Garrett scores by referring to Garrett's conversion table. The percent positions are matched with corresponding Garrett scores from the Garrett table.

**Step 5: Calculation of Mean Garrett Scores:** For each factor, the Garrett scores obtained from all respondents are summed and divided by the total number of respondents to calculate the mean Garrett score.


$$\text{Garrett Mean Score = }\frac{\text{Σ Garrett Score}}{\text{Number of respondents}}$$

**Step 6: Final ranking of factors:** The factors are ranked in descending order based on their mean Garrett scores. The factor with the highest mean score is considered the most important, while the one with the lowest score is considered the least important. The challenge with the highest mean score is considered the most important or severe, followed by the next highest, and so on.

### Formulation of Strategies by selected officials to overcome the challenges

**Figure 3:**
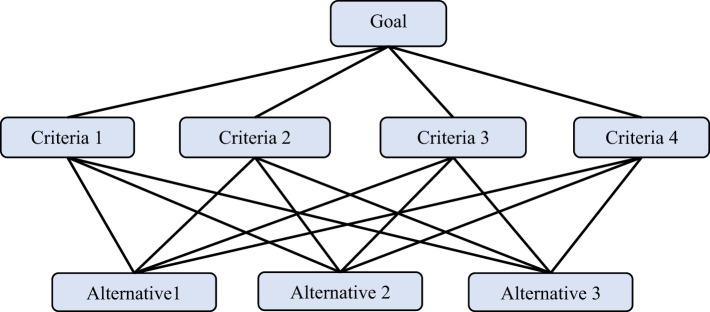
Hierarchical structure of AHP.

To formulate strategic interventions addressing these challenges, the AHP was utilized. AHP, developed by Saaty (1980), is a widely recognized multi-criteria decision-making (MCDM) tool that supports structured decision-making in complex contexts. It was chosen for its capacity to incorporate both qualitative and quantitative criteria by generating weighted priorities through pairwise comparisons. AHP enables systematic evaluation by leveraging expert judgment, making it particularly suitable for socio-developmental studies where diverse and interrelated factors must be prioritized to guide policy and programmatic responses effectively (Mukherjee et al., 2018). Although AHP is based on expert judgements, which may be subjective, efforts were taken to reduce bias by selecting experts with relevant expertise and using consistency tests to ensure logical coherence of the judgements. The AHP model is given in Figure 3.

### Steps and formulas

The AHP involves a series of structured steps to support multi-criteria decision-making.

**Step 1: Problem modeling -** This stage include clearly defining the problem and its objective of the decision. The problem was decomposed into a hierarchy of criteria and sub-criteria, leading to the alternatives being considered. A hierarchical structure was developed, beginning with the overall goal at the top (strategies for empowerment), followed by the relevant criteria at the intermediate level. Then, further establish the alternatives or options at the bottom of the hierarchy as presented in Figure 3.

**Step 2: Construction of Pairwise Matrix-** This step involves constructing a series of pairwise comparison matrices, each of size n × n, representing comparisons among alternatives at a given level with respect to a criterion. Experts compare two elements at a time using Saaty's nine-point scale, where values range from 1 (equal importance) to 9 (extreme importance of one element over another). Reciprocal values are used when the second element is preferred over the first. The study considered a 5 × 5 matrix as follows:


$$\begin{array}{l} \left|\begin{array}{ll} A_{11} & A_{12}\\ B_{21} & B_{22}\\ C_{31} & C_{32}\\ D_{41} & D_{42}\\ E_{51} & E_{52} \end{array}\;\;\;\;\begin{array}{ccc} A_{13} & A_{14} & A_{15}\\ B_{23} & B_{24} & B_{25}\\ C_{33} & C_{34} & C_{35}\\ D_{43} & D_{44} & D_{45}\\ E_{53} & E_{54} & E_{55} \end{array}\right| \end{array}$$

**Step 3: Judgement Scale-** Comparison of the statements was transformed into numerical values reflecting the relative importance of the criteria. A standardized measurement scale (Table 1) is used to conduct these comparisons. The reciprocal values were assigned to ensure consistency within the matrix.

**Table 1:** The pairwise comparison scale for AHP preferences (Saaty, 1986, 1987).

Intensity of importance on an absolute scale	Definition	Explanation
1	Equal importance	Two strategies contribute equally to the objective
3	Moderate importance of one over another	Experience and judgment strongly Favor one strategy over another
5	Essential or strong importance	Experience and judgment strongly Favor one strategy over another
7	Very strong importance	A strategy is strongly favored and its dominance demonstrated in practice
9	Extreme importance	The evidence favoring one strategy over another is of the highest possible order of affirmation
2, 4, 6, 8	Immediate values between the two adjacent judgments	When compromise is needed
Reciprocals	Use reciprocal values for inverse comparisons, that is, 1/3,1/5,1/7,1/9	

**Step 4: Calculation of criteria weights/ priority vector/ scaling factor-** The sum of each column in the matrix was computed, and then each cell value was divided by the sum of the respective column to obtain a normalized value. Criteria weights were determined by taking the average of all the elements in the row and calculating the principal eigenvector corresponding to the maximum eigenvalue (λ_max). The resulting weights provide a basis for prioritization. The complex formulas used in the AHP calculation are given below. The formulas were used for the 5 × 5 matrix as following:

1. Eigen Vector (EV) = GEOMEAN (A11:A15)
2. Total of EV = Sum of all Eigenvector values, [(GEOMEAN (A11:A15) + GEOMEAN (B21:B25) + GEOMEAN (C31:C35) + GEOMEAN (D41:D45) + GEOMEAN (E51:D55))]
3. Weight = Respective Eigen Vector Value/Total of EV. For example, Weight for row 1: GEOMEAN (A11:A15)/Total of Eigen Vectors.
4. Component Eigen Vector = Each Column Element × Weight of Each Row
  For example, for row 1: (A11 × W(row1)) + (A12 × W(row2)) + (A13 × W(row3)) + (A14 × W(row4)) + (A15 × W(row5))
5. λ (Lambda) Max = Sum of Eigen Vector

Step 5: **Consistency Check of Judgments-** The consistency index (CI) measures the degree of logical consistency among pair-wise comparisons. Consistency was evaluated using the maximum eigenvalue (λ_max), Consistency Index (CI), and the consistency ratio (CR). A CR value below a defined threshold indicates acceptable consistency in judgments. For a 5 × 5 matrix used in this study, the random index (RI) value of 1.12 was used to compute the consistency ratio (Soam et al., 2023).


$$\begin{array}{l} \text{CI}=\frac{\left({\text{λ}}_{\max}-n\right)}{\left(n-1\right)}, \end{array}$$

Where *n* is the number of existing items in the judgment matrix problem.

λ_max_ is the maximum eigenvalue

Consistency ratio (CR) indicates the amount of allowed inconsistency, *i.e*., 0.1 or 10%. It is calculated using the following formula:


$$\text{(CR) = }\frac{\text{Consistency Index (CI)}}{\text{Random Index (Size of Matrix)}}$$

The consistency of judgments can be checked by verifying the consistency ratio value. The random index values for matrix sizes are given in Table 2. The current study was a 5 × 5 matrix; therefore, the random index was taken as 1.12 for calculating the consistency ratio for each component of the strategy. CR should be ≤ 0.10. Inconsistency reflects the adjustment needed to improve comparison consistency, but should neither match the original judgment nor be negligible. Ideally, inconsistency should be about 10%—one order of magnitude smaller than the judgment. Lowering it (for example, 1% or 0.1%) would trivialize its importance. However, some inconsistency is necessary to allow new knowledge to influence preferences (Saaty and Vargas, 2006).

**Table 2:** Random Index (RI) for calculating consistency ratio (Saaty and Vargas, 2012, 2013).

Order (size of matrix)	1	2	3	4	5	6	7	8	9	10
Random Index (RI)	0	0	0.58	0.9	1.12	1.24	1.32	1.41	1.45	1.49

## Results

### Challenges faced by Oraon tribal women in their empowerment

**Figure 4:**
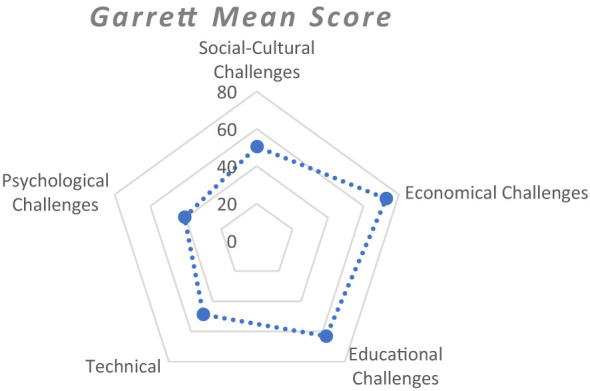
Radar chart showing the severity of challenges.

The challenges faced by women have persisted in tribal communities for ages, and the Oraon tribal women face different obstacles in their daily lives, which impact their overall ability to make the right decision. Therefore, it is important to acknowledge every type of challenge encountered by them. The prioritization of challenges was carried out in a dimension-wise manner to capture the multidimensional nature of women's empowerment. Figure 4 revealed that economic and educational challenges were more severe in the study area. From the Table 3 it evident that the most significant sociocultural issues that women face are the unequal distribution of household responsibilities causes a double burden and undervaluing of their role in household wellbeing with a Garrett mean score of 62.05, lack of nearby standard healthcare facilities affects women's overall health, which limits the efficiency of women's working ability with a Garrett mean score of 60.69 and Early marriage of women affects their ability to make strategic life choices with a Garrett mean score of 45.25. The lack of justified daily wages for women for their agrarian and allied sector work with a Garrett mean score of 60.98, lack of employment opportunities at the village level, especially during off-season with a Garrett mean score of 56.79, and neglecting the financial contributions made by a woman to her family with Garrett mean score of 55.32 are the economic challenges prevalent in their lives.

**Table 3:** Challenges faced by Oraon tribal women in empowerment.

	Challenges	Total score	Garrett mean score	Rank within dimension
Socio-cultural challenges	Dominant role of men in society creates rigid gender roles and limits women's access to decision-making power	17218	43.05	IV
	The unequal distribution of household responsibilities causes a double burden and undervaluing of their role in household well-being	24821	62.05	I
	Lack of nearby standard healthcare facilities affects tribal women's overall health, which limits the efficiency of women's working ability	24275	60.69	II
	Due to traditional beliefs, restrictions are imposed on tribal women's freedom of choice of work and participation in the public sphere	16493	41.23	V
	Early marriage of tribal women affects their ability to make strategic life choices	18101	45.25	III
Economic challenges	Lack of land ownership rights and natural resources, limiting economic independence	17749	44.37	IV
	Lack of freedom to spend their earned income independently	16756	41.89	V
	Lack of justified daily wages for tribal women for their agrarian work and allied sectors	24391	60.98	I
	Lack of employment opportunities at the village level, especially during the off-season	22716	56.79	II
	Economically dependent nature of a tribal women on her husband suppresses her from empowerment	15621	39.05	VI
	Neglecting the financial contributions made by a tribal women to her family	22127	55.32	III
Educational Challenges	Limited access to education due to the vicious cycle of poverty	27519	68.80	I
	Oraon tribal women have the lowest literacy rate due to lack of family support	14803	37.01	V
	Tribal women/girls often drop out of education due to household responsibilities	24185	60.46	II
	Lack of knowledge/interest in the importance of education among tribal women	16450	41.13	IV
	Limited access to smartphones and social media limits women's learning opportunities	14390	35.98	VI
	Girl child is often restricted from education due to gender biases in the household	22417	56.04	III
Technical challenges	Lack of proper outreach of information related to agricultural and nutritional practices, training, and extension services at the village level	21425	53.56	III
	Lack of knowledge, awareness, and functional understanding of improved agricultural practices results in low adoption and stagnant agricultural production	22470	56.18	I
	Lack of access to advisory services due to the mobile gender gap	22053	55.13	II
	Lack of opportunity to access improved technology makes women dependent on labor-intensive traditional farming methods	18894	47.24	IV
	Lack of vocational training and extension programs hinders from gaining additional skills related to agricultural practices	14947	37.37	V
Psychological challenges	Lack of self-efficacy for empowerment	21586	53.97	III
	Household domestic violence and abusive behavior mentally affect women	23546	58.87	I
	Unfavorable attitude of the patriarchal society toward the empowerment of tribal women	21719	54.30	II
	Lack of self-confidence and self-esteem	17717	44.29	IV
	Lack of decision-making ability in the household	14938	37.35	V

Educational challenges faced by Oraon tribal women are limited access to education due to a vicious cycle of poverty with a Garrett mean score of 68.80, tribal women/girls often drop out of education due to household responsibilities with a Garrett mean score of 60.46 and girl child is often restricted from education due to gender biases in the household with a Garrett mean score of 56.04. The most prominent technical challenges encountered by Oraon tribal women are lack of knowledge, awareness, and functional understanding about improved agricultural practices with a Garrett mean score of 56.18 and lack of access to advisory services due to mobile gender gap with a Garrett mean score of 55.13 and lack of proper outreach of information related to agricultural and nutritional practices, training, and extension services at the village level with a Garrett mean score of 53.56. The psychological challenges faced by Oraon tribal women are domestic violence and abusive behavior in the household, which mentally affects women with a Garrett mean score of 58.87, unfavorable attitude of the patriarchal society toward the empowerment of women with a Garrett mean score of 53.30, and lack of self-efficacy with a Garrett mean score of 53.97. The economic challenges with a Garrett mean score of 72.89 and educational challenges with a Garrett mean score of 63.12 were the most serious hindrance factors limiting selected women's empowerment, followed by socio-cultural challenges.

### Strategies to overcome the challenges faced by Oraon tribal women

The challenge-oriented strategies were developed with the matrix ranking data elicited from 40 officials, and their criteria weight and rank are given in Table 4. The findings delineate that mass level awareness campaign on domestic violence, double burden and nutrition programs targeting women and girls (SSC-5) with criteria weight 0.298, promoting women's economic participation through access to financial services and policies fostering inclusion in formal employment sectors (SEC-4) with criteria weight 0.326, increasing investment in tribal education by strengthening infrastructure development, teacher training, curriculum development, and scholarships (SEDC-5) with criteria weight 0.332 were the highest priority strategies to overcome the socio-cultural, economic and educational challenges respectively. Residential training to enhance the awareness and implementation of agricultural and nutritional practices (STC-2) was the highest priority strategy, with a criteria weight of 0.336 for resolving the technical challenges. Engaging men, women, and youth in gender sensitization using folk media, local leaders, and religious figures to support women's roles in development (SPsC-2) was the highest priority strategy with a criteria weight of 0.272 to overcome the psychological challenges.

**Table 4:** Relative weight and rank order of Strategies for Oraon tribal women in empowerment.

Sl. No.	Strategies	Criteria weights	Rank
I	Strategies for socio-cultural challenge (λ_max = 5.351, CI= 0.088, CR= 0.078)		
1	Promote awareness campaigns, education, and community action to prevent early marriage and foster the decision-making power of women (SSC-1)	0.260	II
2	Promote gender-responsive interventions that encourage shared household responsibilities and recognize tribal women's dual role (SSC-2)	0.183	III
3	Encourage tribal women engagement in community life to enhance decision-making ability (SSC-3)	0.102	IV
4	Access to comprehensive reproductive health services through better infrastructure of healthcare facilities (SSC-4)	0.157	V
5	Implement mass-level awareness campaigns on the double burden, and nutrition programs targeting tribal women and girls (SSC-5)	0.298	I
II	Strategies for economic challenge (λ_max = 5.206, CI= 0.052, CR=0.046)		
1	Improve access to land and technology, and support tribal women's cooperatives and self-help groups (SEC-1)	0.249	II
2	Addressing the gender wage gap by promoting pay equity (SEC-2)	0.140	IV
3	Generating employment opportunities with fair and sustainable daily wages (SEC-3)	0.190	III
4	Promoting tribal women's economic participation through access to financial services and policies fostering inclusion in formal employment sectors (SEC-4)	0.326	I
5	Simplifying the loan procedures and providing adequate loan facilities timely (SEC-5)	0.095	V
III.	Strategies for educational challenges (λ_max = 5.254, CI= 0.064, CR= 0.057)		
1	Access to quality education for tribal girls and women at all levels (SEDC-1)	0.227	II
2	Promote gender equality within the family and community for tribal girls' education (SEDC-2)	0.124	IV
3	Promoting skill-based learning (agro-based skills, handicrafts, and traditional art), and community-based vocational training by SHGs (SEDC-3)	0.111	V
4	Enrolment in adult literacy programs to empower illiterate or undereducated tribal women with basic literacy and numeracy skills (SEDC-4)	0.203	III
5	Increase investment in tribal education by strengthening infrastructure development, teacher training, curriculum development, and scholarships (SEDC-5)	0.332	I
IV	Strategies for Technical Challenges (λ_max = 5.422, CI= 0.106, CR= 0.0947)		
1	Improve last-mile outreach with skilled female extension workers, and incorporate Agri-nutrition information into SHG and Anganwadi meetings (STC-1)	0.190	III
2	Residential Training to enhance the awareness and implementation of agricultural and nutritional practices (STC-2)	0.336	I
3	Targeted digital literacy programs and affordable mobile access to tribal women, helping them use mobile advisory services (STC-3)	0.162	IV
4	Vocational training programs in collaboration with KVKs, SHGs, and NGOs related to agriculture practices, promote peer learning, demonstration (STC-4)	0.219	II
5	Dissemination of practice packages on nutrition to improve dietary habits, food preparation, and community-level nutritional awareness (STC-5)	0.093	V
V	Strategies for Psychological Challenges (λ_max = 5.303, CI=0.076, CR= 0.068)		
1	Provide mental health support services tailored to the needs of tribal women through SHGs, NGOs, and community-based support groups (SPsC-1)	0.189	III
2	Engage tribal men, women, and youth in gender sensitization using folk media, local leaders, and religious figures to support women's roles (SPsC-2)	0.272	I
3	Awareness campaigns, workshops, and educational initiatives can be adopted to change attitudes toward domestic violence and mobilize public support for gender equality (SPsC-3)	0.231	II
4	Facilitate mentoring programs through SHGS and promote exposure visits to successful tribal women's enterprises (SPsC-4)	0.169	IV
5	Improve access to justice and support services for tribal women's by strengthening legal aid services (SPsC-5)	0.139	V

### Overall priority scores of strategies

The overall priority scores of strategies were calculated and presented in Table 5. The criteria weight of each strategy of the overall strategies or categories of challenges mentioned in the methodology was multiplied by the criteria weight of the strategy group to get the overall priority scores of each strategy. The result revealed that the priority of economic challenges was maximum at 33.8% followed by educational challenge at 23.5%, socio-cultural challenge at 17.9, technical challenge at 15.5%, and psychological challenge at 9.3%. Thus, the strategic intervention for the Oraon tribal women's empowerment, economic, educational, and socio-cultural challenges, to be taken into account first. The most important economic strategies were SEC-4, that is, opportunities for women's economic participation through access to financial services and policies fostering inclusion in formal employment sectors, with an overall priority value of 0.110. The most important educational strategy was SEDC-5, that is, increased investment in tribal education by strengthening infrastructure development, teacher training, curriculum development, and scholarships, with an overall priority value of 0.078. The most important socio-cultural strategies were SSC-5, that is, implementing a level awareness campaign on the double burden, and nutrition programs targeting women and girls, with an overall priority value of 0.053.

**Table 5:** Overall priority scores of strategies for Oraon tribal women's empowerment.

Strategy Group	Group Priority (Rank)	Strategies	Factor priority within the group	Overall priority of the factor
Socio-cultural Challenges	0.179 (17.9%)	SSC-1	0.260	0.047
		SSC-2	0.183	0.033
		SSC-3	0.102	0.018
		SSC-4	0.157	0.028
		SSC-5	0.298	0.053
Economic Challenges	0.33 (33.8%)	SEC-1	0.249	0.084
		SEC-2	0.140	0.047
		SEC-3	0.190	0.064
		SEC-4	0.326	0.110
		SEC-5	0.095	0.032
Educational Challenges	0.235 (23.5%)	SEDC-1	0.227	0.053
		SEDC-2	0.124	0.029
		SEDC-3	0.111	0.026
		SEDC-4	0.203	0.048
		SEDC-5	0.332	0.078
Technical Challenges	0.155 (15.5%)	STC-1	0.190	0.029
		STC-2	0.336	0.052
		STC-3	0.162	0.025
		STC-4	0.219	0.034
		STC-5	0.093	0.014
Psychological Challenges	0.093 (9.3%)	SPsC-1	0.189	0.018
		SPsC-2	0.272	0.025
		SPsC-3	0.231	0.021
		SPsC-4	0.169	0.016
		SPsC-5	0.139	0.013

## Discussion

The internal characteristics, social norms, and external circumstances and resources all contribute to the empowering process as either facilitators or barriers (Raj et al., 2024). The Oraon tribal women face various daily obstacles that affect their ability to make the right decision. The findings revealed that Oraon tribal women face significant challenges both in the household and at the community level, which are socio-cultural, economic, educational, technical, and psychological. Therefore, it is necessary to empower women in every dimension of empowerment. The most serious challenges were economic barriers, including the lack of justified daily wages, the lack of employment opportunities, and the neglect of the financial contributions made by a woman in the household. This finding is aligned with (Ghosh et al., 2021; Thakur and Kumar, 2024) that though the economic participation rate of the respondents was very high, they had no economic opportunities, and about 95% of the respondents were under the medium income category with high farming experience. Therefore, women's economic participation through access to financial services and policies fostering inclusion in formal employment sectors should be promoted, and income-generating opportunities should be strategized for tribal women. The lack of awareness regarding government schemes aimed at economic empowerment reflects a significant communication and outreach gap. Despite the existence of beneficial programs inadequate dissemination of information, lack of digital access, weak last-mile reach, and insufficient community-level facilitation affect effective implementation. It is crucial to strengthen awareness campaigns, involve grassroots workers like ASHAs and SHGs, and ensure that door-to-door outreach and use of community radio can also enhance reach and impact in tribal areas.

Limited access to education due to the vicious cycle of poverty, and tribal women and girls often drop out of education due to household responsibilities, are the serious educational challenges. The finding is aligned with (Mal and Saikia, 2024) that the status of tribal women in terms of education, work, and health is poor, not just when compared to the status of tribal males, but also the status of women from the general public, making them doubly disadvantaged for being women and their minority group status. There is a wide gap in the rate of literacy between tribal men and women, and there is a need to mobilize their social position through education (Brago et al., 2025). Therefore, the community should be motivated to continue higher studies of women and girls, for which specific fellowships and incentives can be initiated. The theory of empowerment is fostered through education, leadership development, and active participation in decision-making processes, while recognizing the role of both individual and collective agency in challenging social discrimination and advancing gender equality (Sen et al., 2023). Apart from the existing programs like Rajiv Gandhi National Fellowship for ST Students, Kasturba Gandhi Balika Vidyalaya etc., targeted policy-level actions should be undertaken by the government and other organizations to act exclusively on increasing investment in tribal education by strengthening infrastructure development, teacher training, curriculum development, and scholarships, followed by access to quality education for girls and women at all levels, including primary, secondary, and tertiary education.

The main challenges in agricultural participation were their physical weakness, which indicates a lack of proper nutrition (Ghosh et al., 2021). Unequal distribution of household responsibilities causes a double burden, which leads to time trade-offs that negatively affect the consumption of various nutritious foods (Vemireddy and Pingali, 2021), lack of nearby standard healthcare facilities, and early marriage of women, affecting them, making strategic life choices under socio-cultural challenges. Empowerment is a developmental process that necessitates both experience with one's own self-direction and connection to important responsibilities in the social world (Mouchrek and Benson, 2023). Hence, sensitization workshops regarding a balanced diet and a healthy lifestyle can be conducted in the locality itself. Healthy, nutrient-rich recipes can be demonstrated with locally available resources through ICAR initiatives such as Nutri-Sensitive Agricultural Resources and Innovations (NARI), Value Addition and Technology Incubation Centers in Agriculture (VATICA), and Knowledge Systems and Homestead Agriculture Management in Tribal Areas (KSHAMTA). Empowering tribal women in economic, educational, and sociocultural aspects will lead them to the freedom to make decisions in other aspects of their lives.

Oraon tribal women had a lack of functional understanding of improved agricultural practices and a lack of access to advisory services. Hence, residential training programs and participatory programs should be planned and implemented regarding agricultural practices and nutri-sensitive agricultural technologies. Also, vocational training programs should be offered at the grassroots levels in collaboration with Krishi Vigyan Kendra, self-help groups, and non-governmental organizations related to agriculture and nutrition practices, promote peer learning, demonstration, and conduct hands-on farmer field schools. Educating women solves most of the challenges (Nath et al., 2024); women could be educated and supported through mentorship programs to increase their awareness and decision-making power and thereby enhance empowerment. Household domestic violence and abusive behavior mentally affect women, which leads to a lack of self-efficacy, self-confidence, and self-esteem. Therefore, legal education opportunities to prevent domestic violence should be included in the education system to make women aware of their rights. Also, engage men, women, and youth in gender sensitization using folk media, local leaders, and religious figures to support women's roles in development for awareness about women's rights, the value of women's contributions, and the importance of shared responsibilities. It is crucial to offer tribal women chances that will enable them to develop leadership skills for economic self-sufficiency and social change (Thakur and Kumar, 2024).

The Indian government has played a crucial role in promoting women's empowerment through various policies and schemes. Legal safeguards, such as the Panchayats (Extension to Scheduled Areas) Act (PESA) and the Forest Rights Act of 2006 (FRA), are essential for increasing tribal women's participation in local administration and ensuring access to natural resources. Similarly, welfare programs such as Self-Help Groups under the National Rural Livelihoods Mission (NRLM) for fostering economic independence and building social capital, thereby empowering women at both the household and community level by enhancing their capability for agency. Thus, it is very important to integrate women into entrepreneurial activities for more economic gain and decision-making. Because entrepreneurship increases women's ability to make decisions related to innovation, risk, and pro-activities, but despite all that, it demands more attention from them. Tribal women gain empowerment and enhance their intra-household decision-making process as a result of entrepreneurship (Naveen et al., 2023). Integrated Child Development Services (ICDS) contribute significantly to social and nutritional empowerment. MNREGA is the world's largest social welfare program for women's social and economic empowerment. However, insufficient awareness, improper implementation, and structural gender barriers limit their (schemes) potential influence and benefits at the grassroots level. Thus, Oraon tribal women's empowerment depends not only on individual or community-level measures but also on the effective implementation and convergence of existing policies and programs. While these initiatives have had a positive impact, there is a need for more robust implementation, monitoring, and evaluation to ensure that the benefits reach all women, particularly Oraon tribal women and marginalized communities. In this regard, Longwe's framework for gender analysis can be used to assess the effectiveness and impact of these governmental programs on achieving gender equality and empowerment and to implement necessary corrective measures. For the holistic empowerment of Oraon tribal women, a women-led development approach should be adopted, wherein women are recognized as central agents of change and actively engaged in planning, decision-making, implementation, and monitoring of development interventions, thereby strengthening their socio-economic agency, leadership capacities, and long-term sustainability of outcomes. To achieve true women's empowerment, it is essential to address the underlying socio-cultural norms that perpetuate gender inequality and to create an enabling environment where women can thrive and contribute fully to society. The inclusion of the above-asserted challenges and strategies in the programs and policy formulation and implementation can be a great step to overcome the challenges faced by tribal women.

## Conclusion

Self-reliant India is the need of the hour, and it will not be possible to achieve without empowering women. In a true sense, women-led development will open the path to a self-reliant India. Therefore, the study addressed the most prominent challenges prevalent in the Oraon tribal community. Lack of justified daily wages, lack of employment opportunities, neglect of financial contributions made by a woman, limited access to education, high dropout of education, domestic violence, double burden, lack of nearby standard healthcare facilities, and lack of functional understanding of technologies hinder tribal women's strategic life choices. More attention should be given to the economic and educational aspects of empowerment to solve most of the challenges altogether. Enhancing women's economic participation and access to resources, increasing investment in tribal education and access to quality education, and residential training and workshops can lead to holistic empowerment of tribal women. The findings of the study can be incorporated into the extension programs, awareness campaigns, and policy formulation, thereby helping India achieve SDG 5, that is, gender equality, and become self-reliant.

## Limitations

The geographic focus was restricted to Jharkhand and Chhattisgarh, which may limit the results' general relevance to other locations or tribal situations. The cross-sectional design captures responses at a single point in time and does not allow for causal interpretations or analysis of temporal changes. Furthermore, relying on self-reported data may introduce recall and social desirability biases. Although consistency measures and structured procedures were employed to reduce bias, some degree of subjectivity in expert judgments cannot be eliminated completely.

## Data Availability

The raw data supporting the conclusions of this article will be made available by the authors, without undue reservation.
